# Inequities in dementia diagnosis and care: insights from a universal health care system

**DOI:** 10.1002/alz70860_107449

**Published:** 2025-12-23

**Authors:** Claire Godard‐Sebillotte, Sanjna Navani, Wang Xia, Genevieve Arsenault‐Lapierre, Nadia Sourial, Amélie Quesnel‐Vallée, Louis Rochette, Victoria Massamba, Isabelle Vedel

**Affiliations:** ^1^ McGill University, Montreal, QC, Canada; ^2^ University of Montreal, Montreal, QC, Canada; ^3^ INSPQ, Quebec, QC, Canada; ^4^ Institut national de santé publique du Québec (INSPQ), Quebec, QC, Canada

## Abstract

**Introduction:**

Burgeoning literature reports inequities in dementia diagnosis and care; evidence from a universal health care system using a comprehensive set of indicators and an intersectional approach is lacking.

**Methods:**

We conducted a repeated yearly cohort study of community‐dwelling people in Quebec with incident dementia (2000‐2017). We described dementia diagnosis and 23 indicators of health service use and mortality across levels of material deprivation, derived from a validated ecological index based on the average income, employment, and education of residential neighborhoods. We then conducted preliminary description of patterns of primary care use across material deprivation and a neighborhood racialization index, derived from a census question around self‐identification as visible minority.

**Results:**

Of the 193,834 community‐dwelling people with a new diagnosis of dementia, around 20% belonged to each material deprivation category. Age‐standardised rates of 15/23 indicators differed across SES. People from most deprived areas had more hospitalisations, emergency department visits, potentially inappropriate medication prescriptions, and higher 1‐year mortality, though they had higher care continuity. Conversely, rates were comparable across groups for the prescription of dementia‐specific medications, and primary care visits. Patterns of primary care use varied across material deprivation and neighborhood racialization; with higher use in wealthiest neighborhoods, the highest use being in the most racialized ones.

**Discussion:**

Despite global findings of higher dementia incidence in lower SES, we found similar incidence across SES. These findings indicate that there is likely severe under‐diagnosis of dementia in more materially deprived neighborhoods. Stark differences across SES in service use by people with dementia may indicate different health needs and/or allude to pervasive health inequities. An intersectional lens allows for a nuanced description of inequalities. These results can inform policies to offer equitable, appropriate, and needs‐based care to all people living with dementia.

